# Experiences on recruitment and retention of volunteers in the first HIV vaccine trial in Dar es Salam, Tanzania - the phase I/II HIVIS 03 trial

**DOI:** 10.1186/1471-2458-13-1149

**Published:** 2013-12-09

**Authors:** Muhammad Bakari, Patricia Munseri, Joel Francis, Eric Aris, Candida Moshiro, David Siyame, Mohamed Janabi, Mary Ngatoluwa, Said Aboud, Eligius Lyamuya, Eric Sandström, Fred Mhalu

**Affiliations:** 1 Muhimbili University of Health and Allied Sciences, P.O. Box 65001, Dar es Salaam, Tanzania; 2 National Institute for Medical Research, Muhimbili Centre, Dar Es Salaam, Tanzania; 3 Muhimbili National Hospital, Dar es Salaam, Tanzania; 4 Tanzania Police Force, Dar es Salaam, Tanzania; 5 Karolinska University Hospital, Stockholm, Sweden

## Abstract

**Background:**

Eventual control of HIV/AIDS is believed to be ultimately dependent on a safe, effective and affordable vaccine. Participation of sub-Saharan Africa in the conduct of HIV trials is crucial as this region still experiences high HIV incidences. We describe the experience of recruiting and retaining volunteers in the first HIV vaccine trial (HIVIS03) in Tanzania.

**Methods:**

In this trial enrolled volunteers from amongst Police Officers (POs) in Dar es Salaam were primed with HIV-1 DNA vaccine at months 0, 1 and 3; and boosted with HIV-1 MVA vaccine at months 9 and 21. A stepwise education provision/sensitization approach was employed to eventual recruitment. Having identified a “core” group of POs keen on HIV prevention activities, those interested to participate in the vaccine trial were invited for a first screening session that comprised of provision of detailed study information and medical evaluation. In the second screening session results of the initial assessment were provided and those eligible were assessed for willingness to participate (WTP). Those willing were consented and eventually randomized into the trial having met the eligibility criteria. Voluntary participation was emphasized throughout.

**Results:**

Out of 408 POs who formed the core group, 364 (89.0%) attended the educational sessions. 263 out of 364 (72.2%) indicated willingness to participate in the HIV vaccine trial. 98% of those indicating WTP attended the pre-screening workshops. 220 (85.0%) indicated willingness to undergo first screening and 177 POs attended for initial screenings, of whom 162 (91.5%) underwent both clinical and laboratory screenings. 119 volunteers (73.5%) were eligible for the study. 79 were randomized into the trial, while 19 did not turn up, the major reason being partner/family advice. 60 volunteers including 15 females were recruited during a one-year period. All participated in the planned progress updates workshops. Retention into the schedule was: 98% for the 3 DNA/placebo vaccinations, while it was 83% and 73% for the first and second MVA/placebo vaccinations respectively.

**Conclusion:**

In this first HIV vaccine trial in Tanzania, we successfully recruited the volunteers and there was no significant loss to follow up. Close contact and updates on study progress facilitated the observed retention rates.

**Trial registration numbers:**

ISRCTN90053831 ISRNCT01132976 and ATMR2009040001075080

## Background

Despite multiple preventive measures to control HIV transmission, the pandemic still claims a substantial number of lives and causes significant morbidity worldwide. HIV/AIDS still remains a disease of public health importance especially in sub-Saharan Africa. According to the UNAIDS report of 2012 the number of people living with HIV at the end of 2011 was estimated to be 35.9 million while the number of people (adults and children) acquiring HIV infection in 2011 was 1.8 million [1.6 million – 2.0 million] [1]. Although the availability of HAART has dramatically improved the quality of life and life expectancy of people living with HIV and AIDS, it is generally believed that the ultimate control of this pandemic depends upon the availability of a safe, effective and affordable HIV vaccine, as it has happened with other viral borne diseases of public health importance such as small pox [2].

Participation of sub-Saharan Africa in the conduct of HIV trials is crucial; since this is the region that is still affected by high HIV incidence [1]. However, recruitment of volunteers in HIV vaccine trials might be a challenge due to a number of reasons including the fact that misconceptions are rampant in such communities [3]. The misconceptions are most likely due to limited overall societal knowledge on vaccine trials as well as minimal exposure to such trials.

Laboratory reference values adopted from the developed countries can also be a limiting factor. Recruitment experiences in Uganda revealed that of the 223 volunteers screened, 69 (31%) were excluded due to haematologic abnormalities [4]. If local reference ranges had been employed, 83% of those screened out due to these abnormalities could have been included in the study, drastically reducing workload and cost associated with the screening process. It was also proposed that toxicity tables used in vaccine and drug trial safety evaluations may need adjustment as some clinical reference ranges determined in the study overlapped with grade 1 and grade 2 adverse events [4].

Engaging in risky sexual behaviors by the would-be trial volunteers could be another factor. In South Africa it was observed that male volunteers engaged more in risky sexual behaviors than female volunteers that included casual multiple sexual partners. This necessitated more risk-reduction counseling in a population that was being prepared for participation in early HIV vaccine trial [5].

There have also been reports documenting the difficulty in recruiting females in HIV vaccine trials despite the fact that women are the most vulnerable and most affected by HIV. Reasons cited include fear of acquiring HIV-infection from the vaccine, testing positive for HIV antibodies, effect of the vaccine upon future pregnancies, and being mistakenly viewed as being HIV infected [6].

Challenges in recruiting volunteers are not restricted to resource-constrained countries, and thus various strategies have been employed in recruiting volunteers into HIV vaccine trials. In the UK, the most successful recruiting strategies for participation in two Phase II prophylactic HIV vaccine (PHV) trials targeted organizations dealing with HIV, health or social issues and were directed to large audiences through the mass media. However, circulated e-mails and word of mouth were the most resource-effective approaches. Group discussions and the collection of a pool of potential volunteers were much less effective than one-to-one discussions and immediate screening after recruitment. These findings were subsequently utilized in devising key recommendations to assist PHV trial teams who were planning subsequent studies [7].

In Thailand, correlates of being screened and recruited into a phase I/II preventive HIV-1 vaccine trial included social demographic variables, motivation for interest in the trial, and factors related to communication and contact. Participants were recruited at two sites through varied methods. The majority of individuals pre-screened reported altruistic motives for interest in the trial, and blood donors emerged as a group that may have been particularly altruistic. Findings indicated site differences in attrition during recruitment and screening, but not in enrollment into the vaccine trial. Blood donation and willingness to be contacted by phone at home were significantly related to making and keeping screening appointments [8].

Preparations towards HIV vaccine trials in Tanzania begun in 1989 within the Tanzania and Sweden (TANSWED) HIV Programme, funded by Sida/SAREC (Swedish Government’s Research Cooperation with Developing Countries) that included capacity building and preparation of a potential cohort of Police Officers (POs). In 2002, a European Union (EU) funded HIV Vaccine Immunogenicity Study (HIVIS) project was initiated to develop and try out a candidate HIV-DNA prime followed by HIV-1 MVA boost vaccine and to further build capacity in conducting HIV vaccine trials in Tanzania.

The Phase I/II HIV Vaccine Immunogenicity Study (HIVIS-03) performed between February 2007 and February 2010 was the first HIV vaccine trial to be conducted in Dar es Salaam, Tanzania. It was a double blinded, placebo controlled vaccine trial of a candidate vaccine consisting of priming with multi-gene, multi-clade (A, B, C) HIV-1 DNA and boosting with HIV-1 MVA (CRF01 subtypes A, E). The trial enrolled 60 volunteers from the Police Force in Dar es Salaam. The cohort was chosen as there had been a prior research contact, (through a Sida/SAREC funded TANSWED HIV Programme) with it that led to the establishment of HIV prevalence and incidence in the Force [9,10]. Additionally, they were chosen by taking advantage of the fact that Police Officers (POs) were from an organized institution that would have allowed easier long-term follow up. It has been reported that the vaccine in the HIVIS-03 trial was safe, and induced broad and potent immune responses against HIV-1 [11].

We hereby describe our experiences in volunteer recruitment and their retention in the HIVIS03 trial.

## Methods

The recruitment process involved multiple educational sessions at different stages that included the following:

### Educational sessions at Police stations & formation of the POs “Core group”

These were conducted between January 2005 and April 2007 at the respective police stations in Dar es Salaam, Tanzania. The sessions included a briefing of the current situation and epidemiology of HIV worldwide and in Tanzania, as well as the magnitude of HIV in the Police Force in Dar es Salaam from previous research conducted within the Police Force. This was then followed by general information on basic concepts of HIV vaccine trials, and the need for participation of resource constrained countries. The study research assistants obtained personal contact information of POs who were willing to be members of the “core group” to actively participate in anti-HIV activities.

### Sensitization of the core group members

Police Officers who were interested to be members of the core group received further educational sessions that again included a briefing of the current situation and epidemiology of HIV worldwide and in Tanzania, as well as in the Police Force in Dar es Salaam. This was then followed by information on the HIVIS 03 trial including the inclusion and exclusion criteria, the details of the vaccines to be administered, the possible adverse reactions, mode of the vaccines administration, study schedule and duration. It was particularly emphasized that participation in the trial was voluntary. Participants were given ample time to ask questions, and adequate answers were provided. The study Research Assistants obtained personal contact information of the POs who were interested in further information about the present study. This information was obtained through a one page sheet that asked if they were willing to be provided with further detailed information on the trial, and if they were willing to be contacted further, and if so, their names and contact details.

### Pre-screening workshops

Police Officers who were interested in further details of the HIV vaccine trial were thereafter contacted and invited to one of the eight pre-screening workshops, conducted between 25th November 2006 and 10th November 2007, whereby detailed information about the study was provided. The information included the risk and benefits of participating in the HIVIS 03 trial and study related procedures. Following the briefing session, the study team obtained contact information of the POs who expressed interests and were willing to undergo screening. The Police Officers who were interested were thereafter invited to the clinical trial site at the Muhimbili National Hospital (MNH) in Dar es Salaam for screening.

### First screening

Screening took place between February and November 2007 at the clinical trial site. Every participant was offered an information sheet that described the study and related procedures. Information on risk and benefits of participating was also provided. The participants were then allowed to ask questions and they were provided with answers by the study Nurse/Doctor. The participants were there after requested to sign an informed consent if they were willing to participate in the trial. All participants were pre and post-test counseled for HIV followed by an abbreviated risk-assessment for HIV including prior history of sexually transmitted infections and risky sexual practices.

Thereafter the study medical Doctor took a detailed history and conducted a clinical examination on the participants and the information obtained was documented in a structured case report form (CRF). The participants were then asked to provide blood and urine samples for laboratory screening tests. The laboratory tests included urinalysis, HIV, syphilis and Hepatitis B surface antigen, clinical chemistry (bilirubin; total and direct, ALT, creatinine and random blood glucose); hematology (total white blood cells count, granulocyte count, lymphocyte count, hemoglobin, and platelet count) and CD4^+^ and CD + 8 T-Lymphocyte counts. In addition, all female volunteers underwent urine pregnancy testing. Laboratory reference values for hematology and clinical chemistry were initially adopted from the Mbeya Medical Research Programme (MMRP) [12]. Subsequently these were modified, especially with fasting blood glucose level to be based on WHO criteria. Reference values for CD4 count were from the Dar es Salaam population as determined earlier [13].

Following specimen collection all participants were reimbursed for their time and travel to an amount approved by the ethics committee of Tanzanian Shillings 20,000/=, that was equivalent to about 20 USD. The participants were then requested to return two weeks later to attend the second screening visit and receive the initial screening test results.

### Second screening

In the second screening visit the participants received all the laboratory results for specimens collected in the first visit and again their willingness to proceed with the trial was assessed. The study Doctor thereafter assessed for the inclusion and exclusion criteria and all volunteers who fulfilled the inclusion criteria for the study, including not testing HIV-positive, were randomized to receive either the HIV-1 DNA vaccine or a placebo. The criteria for inclusion into the trial have been reported earlier [11].

Randomized participants were thereafter offered appointment dates for their first vaccination (enrolment) within one month of the initial screening.

### Post-enrolment procedures

Enrolled volunteers received 3 HIV-1 DNA or placebo doses (as priming vaccines) at months 0, 1 and 3, by a needle less device, Biojector, either intramuscularly or intradermally. This was followed by boosting with 2 intramuscular injections of HIV-1 MVA-CMDR or placebo at months 9 and 21.

As per protocol, volunteers had to attend a total of 24 scheduled visits. They were assessed for safety as well as immunogenicity two weeks after each HIV-1 DNA or HIV-1 MVA vaccination. These were also performed at 4 and 24 weeks after the HIV-MVA immunizations. Details of the vaccination schedules have been described earlier [11].

To ensure retention, the study team had close contact with the volunteers through mobile phone communications, physical visits to their places of work or domicile when necessary, as well as holding three monthly meetings with all volunteers where study progress and challenges were mutually discussed.

### Data analysis

Data were analyzed using SPSS version 18.0 software. Proportions or medians (interquartile range [IQR]) were calculated. Comparison of proportions was done by Chi-square or Fisher’s exact test where applicable. A two-sided p-value of less than 0.05 was considered statistically significant.

### Ethics

The HIVIS-03 study protocol received ethical approvals from the National Ethics Committee at the National Institute for Medical Research (NIMR) and the local Institutional Review Board of Muhimbili University of Health and Allied Sciences (MUHAS). The Tanzania Food and Drug Authority (TFDA) approved the importation of both the DNA and MVA vaccines and for their use in healthy adults in the trial.

All screened volunteers and those randomized into the trial signed informed consent form after having read through the study information sheet.

Voluntary participation was emphasized at all levels of Police Force hierarchy. All volunteers found to have HIV infection and other medical problems during screening were referred for appropriate care at the MNH.

## Results

### Educational sessions, screening and enrolment

**Figure 1:**
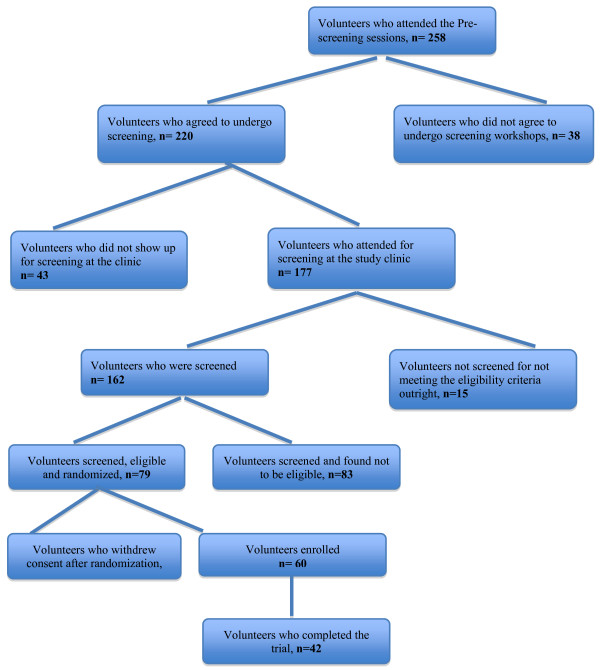
Flow Chart.

A “core-group” of 408 POs interested in the fight against HIV and AIDS was formed from a repeat HIV incidence study supported by Sida/SAREC under the TANSWED HIV Programme. From these, a total of 346 (84.8%) volunteers participated in the sensitization educational sessions. Of these, 258 (72.8%) attendees were willing to participate in the vaccine trial and hence were ready to be offered further study details, and of these 220 (87.3%) volunteers attended the pre-screening sessions. Further, 177 (80.5%) volunteers were willing and attended the clinic to be screened, of whom 48 (27.1%) were female. Fifteen of these were not eligible outright (reasons being pregnancy, age above 40 years, and desire to have children within two years). The remaining 162 were screened. A summary of volunteers’ participation in the HIVIS-03 trial is shown in Figure 1 and Table 1.

**Table 1:** Participation of volunteers in the HIVIS-03 trial

**Volunteers who:**	**Males (%)**	**Females (%)**	**Total (100%)**
Attended for Screening	128 (72.3%)	49 (27.7%)	177 (100%)
Screened with available Lab test results	119 (73.5%)	43 (26.5%)	162 (100%)
Randomized	57 (72.2%)	22 (27.8%)	79 (100%)
Exited after Randomization	12 (63.2%)	7 (36.8%)	19 (100%)
Enrolled & received 1st DNA/Placebo vaccination	45 (75%)	15 (25%)	60 (100%)
Screening: Enrolment	2.8:1	3.3:1	3.0:1

Of 162 volunteers screened between January 2007 to November 2007, 79 were found to be eligible and were subsequently randomized. Volunteers above the age of 40 were screened initially, but following the findings from a similar trial in Stockholm (HIVIS-01/02) which showed that those with ages above 40 years had poor immune responses, the age criterion in Dar es Salaam was changed to include only those between the ages of 18 and 40 years [14].

A comparison of the baseline socio-demographic characteristics of volunteers who were screened and eventually enrolled is summarized in Table 2. Of the enrolled volunteers, 36 (60%) had at least secondary education and 37 (62%) were not married.

**Table 2:** Baseline characteristics of the screened Police volunteers

**Characteristic**	**Attended for screening**	**Randomized**	**Enrolled**
Number	177	79	60
Male : Female	126:51	57:22	45:15
Age Range (years)	20-52	19-47	20-47
Median Age (IQR)*	28(24–35)	26	28 (25–35)
Female	26(22–36)	27.5	25 (23–36)
Male	28 (25–35)	26	28 (25–32)
Education, n (%)			
*Primary*	55 (31.6)	8	16(26.7)
*Secondary*	98 (56.3)	58	36 (60.0)
*Post-secondary*	21 (12.1)	13	8 (13.3)
Marital Status, n (%)			
*Single*	105 (60.3)	51	37(61.7)
*Married/cohabiting*	68 (39.7)	28	23(38.3)

*IQR = interquartile range.

The reasons for ineligibility after screening are shown in Table 3. Of the 79 volunteers who were eventually randomized, 19 were exited for various reasons (Table 4). Seven (36.8%) were female, and 12 (63.2%) were male. The reasons have been published earlier [3]. In brief, the main reason for screen out was abnormal laboratory values during screening which accounted for about 46% of the reasons for screen out.

**Table 3:** Reasons for screen out of volunteers in the HIVIS-03 trial (n = 83)

**Reason**	*Number	**%**
Abnormal laboratory values	41	46.1
Age above 40 years	10	11.2
Had multiple sexual partners	5	5.6
Elevated Blood Pressure	6	6.7
Parents/relatives do not support participation	7	7.9
Desire for having children in coming 2 years	5	5.6
Pregnancy	4	4.5
Did not show up for results/planned visit	6	6.7
Declined to repeat test	2	2.2
Undecided	2	2.2
Other reasons	10	11.2

*Multiple reasons possible in a single individual.

**Table 4:** Retention of HIVIS-03 trial volunteers

**Volunteers who were:**	**Total**	**Males**	**Females**	p-value*
	n (%)	n (%)	n (%)	
Randomized	79	57	22	
Exited after Randomization	19 (24.1%)	12 (21.1%)	7 (31.8%)	0.32
				
Enrolled	60	45	15	
***Vaccination Visits***				
Received 1st DNA/Placebo	60 (100%)	45 (100%)	15 (100%)	-
Received 2nd DNA/Placebo	60 (100%)	45 (100%)	15 (100%)	-
Received 3rd DNA/Placebo	59 (98.3%)	44 (97.8%)	15 (100%)	0.99
Received 1st MVA/Placebo	50 (83.3%)	41 (91.1%)	9 (60%)	0.02
Received 2nd MVA/Placebo	42 (70%)	37 (82.2%)	5 (33.3%)	0.002
***Safety and Immunogenicity visits***				
2 weeks post 2nd DNA/Placebo	60 (100%)	45 (100%)	15 (100%)	-
2 weeks post 3rd DNA/Placebo	59 (98.3%)	44 (97.8%)	15 (100%)	0.99
2 weeks post 1st MVA/Placebo	50 (83.3%)	41 (91.1%)	9 (60%)	0.02
8 weeks post 1st MVA/Placebo	50 (83.3%)	41 (91.1%)	9 (60%)	0.02
24 weeks post 1st MVA/Placebo	42 (70%)	37 (82.2%)	5 (33.3%)	0.002
2 weeks post 2nd MVA/Placebo	42 (70%)	37 (82.2%)	5 (33.3%)	0.002
4 weeks post 2nd MVA/Placebo	42 (70%)	37 (82.2%)	5 (33.3%)	0.002
24 weeks post 2nd MVA/Placebo	40 (66.7%)	35 (77.8%)	5 (33.3%)	0.002

*p-values from Chi square or Fisher’s exact test.

The first volunteer was enrolled on 27th February 2007, while the last volunteer was enrolled on the 26th February 2008. Of the 60 volunteers enrolled into the study, 15 (25%) were female. The overall median (range) age of volunteers was 28 (20–47) years. Recruitment up to the last female volunteer spanned over a period of 12 months.

### Retention

The 2nd MVA/Placebo vaccinations began on 3rd February 2009, and vaccinations were completed in July 2009. Table 4 summarizes the retention of volunteers into the trial. It has to be appreciated that of the enrolled 60 volunteers, 42 (70%) completed the trial with all the five vaccinations. Noteworthy is the fact that there was a statistically significant difference in the proportion of female volunteers who continued with the 1st and 2nd MVA vaccinations as well as those who came up for non-vaccination visits when compared to male participants.

A total of 18 volunteers including 10 out of 15 females were discontinued from further vaccinations at different stages of the trial due to various reasons. These were either investigator initiated (in 11 volunteers), or volunteer initiated (in 7 volunteers). Reasons for discontinuation among the 11 volunteers in the first group were: seizure disorder (1 volunteer), sickle cell disease (1 volunteer), pregnancy (3 volunteers), hypertension (1 volunteer), imprisonment (1 volunteer), elevated serum bilirubin (1volunteer), benign ovarian tumor (1 volunteer), HIV infection (1 volunteer), and uticaria (1 volunteer). Reasons for the 7 volunteers who withdrew from the study on their own were: relocation (1 volunteer) and social reasons (6 volunteers). Hence the most common reasons for discontinuation were medical, and all such volunteers were followed up for safety till the end of the study in accordance with the study protocol.

## Discussion

This study describes the recruitment and retention experiences in the first HIV vaccine trial in Dar es Salaam, Tanzania. We have shown that with extensive prior interactions and targeted communication, it was possible to recruit all the required 60 volunteers into a Phase I/II HIV vaccine trial in a resource constrained setting, and in an environment with no prior HIV vaccine clinical trial experience. It certainly was an arduous task that required a whole year to enroll the sixty volunteers.

Challenges of recruiting volunteers have been reported in several settings and a number of factors are known to influence the willingness to participate (WTP) in HIV vaccine trials. A study in India among men having sex with men (MSM) concluded that pervasive familial, community and social-structural factors characteristic of the Indian socio-cultural context could have been responsible for complicating the individual-focused approaches to WTP and thereby constraining the effectiveness of interventions to support recruitment and retention in HIV vaccine trials [15]. Other studies have documented factors such as engaging in risky sexual behavior by potential volunteers [5]. On the other hand, among women, fear of contracting HIV from the vaccine, testing positive for HIV, the effect of the vaccine upon future pregnancies and mistakenly viewed as being HIV infected were regarded as important factors affecting their recruitment [6]. It was not surprising therefore that in this HIVIS-03 trial, being the first such trial in the country, recruitment had to take such a long time.

Recruitment of volunteers in this trial involved multilevel educational approaches that essentially emphasized the role of communication and engagement with the community in recruiting volunteers into HIV vaccine trials. This is true in resource limited countries like Tanzania, but is also important even in a developed country setting [16]. A survey of individuals from areas of high poverty and high HIV prevalence in Atlanta USA that employed multilevel modeling techniques concluded that community-level factors do facilitate participation in HIV vaccine research independent of both individual- and social/organizational-level factors [17].

It has to be emphasized that the POs participated in this trial as individuals, and there was no coercion from police peers. This fact was emphasized at multiple levels of the Police hierarchy. The PO’s were chosen based on the fact that we had a prior HIV research experience with them whereby we had also shown that the cohort was suitable for HIV vaccine trials [9]. Additionally we wanted to take advantage of the fact that volunteers from such an institutionalized organization will facilitate their follow-up. Use of volunteers from institutionalized organizations for ease of follow up has been found to be desirable as reported in Nigeria with University students [18].

Abnormal laboratory values and un-supporting family members have been the main reasons for ineligibility in this study. In Kenya it has been reported that over 61% of the screening exclusions in clinically healthy people were due to laboratory abnormalities and hence calling for the generation of laboratory reference ranges from local populations that can be used in the conduct of clinical trials to avoid unnecessary exclusion of willing participants and to avoid over-reporting of adverse events for enrolled participants [19]. In this effort, a large scale study from multiple sites in Africa showed that detection of hepatitis B surface antigen was the commonest laboratory abnormality among potential volunteers for clinical trials [20].

It was a challenge to recruit and retain female volunteers in this trial, partly contributed to by the fact that female Police Officers in the cohort were a minority. Additionally, the study requirement that a volunteer should not conceive during the entire study duration might also have contributed to the dismal recruitment and retention rates of female volunteers. This is supported by the observation that a number of female volunteers became pregnant during the study and were thus unable to complete the study protocol. It follows that for future trials enrolment of volunteers should extend beyond this population.

It was of note that 24.1% of volunteers exited after randomization. The possible reasons for such an event have been reported elsewhere [3]. As reported earlier, this calls for expanding the HIV vaccine trial education to the general population from the onset of the trial design.

The fact that 70% of the enrolled volunteers completed such a protracted trial is worth reckoning as it implies that indeed retention was excellent after enrolment. It is believed that this has been greatly contributed by the excellent relationship that was built between the participants and the study staff.

### Study limitation

The reported findings relate to a specific trial and a specific population and hence may not be generalized.

## Conclusion

Although this was the first HIV vaccine trial in Tanzania, we successfully recruited study volunteers into the trial and there was no significant loss to follow up after recruitment. Close contact and frequent educational sessions to the volunteers with updates on study progress facilitated the observed retention rates to the vaccination schedule.

### Recommendation

Recruitment and retention of volunteers has been possible, but it is an effort that calls for extensive educational sessions and a very close interaction with the community.

## Competing interests

All authors declare no conflict of interest in this work.

## Authors’ contributions

MB, ES, FM and MJ conceived the initial idea to conduct the study. FM, MB, JF, DS, MJ, EA, MN, SA, EL were involved in study implementation as well as finalization of the manuscript. CM and ES were instrumental in data management including statistical analyses. MB, JF, and PM wrote the initial draft of the manuscript and oversaw its finalization. All authors read and approved the final manuscript.

## Pre-publication history

The pre-publication history for this paper can be accessed here:

http://www.biomedcentral.com/1471-2458/13/1149/prepub
